# Hepatocellular Carcinoma Screening in a Contemporary Cohort of At-Risk Patients

**DOI:** 10.1001/jamanetworkopen.2024.8755

**Published:** 2024-04-29

**Authors:** Darine Daher, Karim Seif El Dahan, Nicole E. Rich, Nabihah Tayob, Vincent Merrill, Daniel Q. Huang, Ju Dong Yang, Anand V. Kulkarni, Fasiha Kanwal, Jorge Marrero, Neehar Parikh, Amit G. Singal

**Affiliations:** 1 Division of Digestive and Liver Diseases, Department of Internal Medicine, University of Texas Southwestern Medical Center, Dallas; 2 Department of Data Science, Dana-Farber Cancer Institute, Boston, Massachusetts; 3 Department of Medicine, Yong Loo Lin School of Medicine, National University of Singapore, Singapore, Singapore; 4 Department of Internal Medicine, Cedars Sinai Medical Center, Los Angeles, California; 5 Department of Hepatology and Liver Transplantation, AIG Hospitals, Hyderabad India; 6 Section of Gastroenterology and Hepatology, Department of Medicine, Baylor College of Medicine, Houston, Texas; 7 Michael E. DeBakey Veterans Affairs Medical Center, Houston, Texas; 8 Department of Internal Medicine, University of Pennsylvania, Philadelphia; 9 Department of Internal Medicine, University of Michigan, Ann Arbor

## Abstract

**Importance:**

Cohort studies demonstrating an association of hepatocellular carcinoma (HCC) screening with reduced mortality are prone to lead-time and length-time biases.

**Objective:**

To characterize the clinical benefits of HCC screening, adjusting for lead-time and length-time biases, in a diverse, contemporary cohort of at-risk patients.

**Design, Setting, and Participants:**

This retrospective cohort study of patients with HCC was conducted between January 2008 and December 2022 at 2 large US health systems. Data analysis was performed from September to November 2023.

**Main Outcomes and Measures:**

The primary outcome was screen-detected HCC, defined by abnormal screening-intent abdominal imaging or α-fetoprotein level within 6 months before diagnosis. Cox regression analysis was used to characterize differences in overall survival between patients with screen-detected and non–screen-detected HCC; lead-time and length-time adjustments were calculated using the Duffy parametric formula.

**Results:**

Among 1313 patients with HCC (mean [SD] age, 61.7 [9.6] years; 993 male [75.6%]; 739 [56.3%] with Barcelona Clinic Liver Cancer stage 0/A disease), HCC was screen-detected in 556 (42.3%) and non–screen detected in 757 (57.7%). Patients with screen-detected HCC had higher proportions of early-stage HCC (393 patients [70.7%] vs 346 patients [45.7%]; risk ratio [RR], 1.54; 95% CI, 1.41-1.70) and curative treatment receipt (283 patients [51.1%] vs 252 patients [33.5%]; RR, 1.52; 95% CI, 1.34-1.74) compared with patients with non–screen-detected HCC. The screen-detected group had significantly lower mortality, which persisted after correcting for lead-time bias (hazard ratio, 0.75; 95% CI, 0.65-0.87) in fully adjusted models. Both groups had similar tumor doubling times (median [IQR], 3.8 [2.2-10.7] vs 5.6 [1.7-11.4] months) and proportions of indolent tumors (28 patients [35.4%] vs 24 patients [38.1%]; RR, 0.93; 95% CI, 0.60-1.43). Adjustment for length-time bias decreased survival estimates, although 3-year and 5-year survival for patients with screen-detected HCC remained longer than that for patients with non–screen-detected HCC.

**Conclusions and Relevance:**

The findings of this cohort study suggest that HCC screening is associated with reduced mortality even after accounting for lead-time and length-time biases. However, these biases should be considered in future studies.

## Key Points

**Question:**

Is screening for hepatocellular carcinoma (HCC) in patients with cirrhosis associated with a survival benefit after accounting for lead-time and length-time biases?

**Findings:**

In this cohort study of 1313 patients, only 42.3% of cases of HCC were detected by screening. Detection by screening was associated with improved early-stage detection and survival, which persisted after adjusting for lead-time and length-time biases.

**Meaning:**

These findings suggest that HCC screening is associated with reduced mortality, even after accounting for lead-time and length-time biases, and remains an important target for interventions to increase utilization.

## Teaser

This cohort study examines the clinical benefits of screening for hepatocellular carcinoma in at-risk patients, adjusting for lead-time and length-time biases.

## Introduction

Hepatocellular carcinoma (HCC) is the fourth leading cause of cancer-related death worldwide.^1,2^ One of the main determinants of prognosis is tumor stage and eligibility for curative therapy. Patients with early-stage HCC have 5-year survival rates approaching 70% with curative treatments, compared with a median survival of 1 to 2 years after palliative therapies for those with more-advanced tumor burden.^3^ Guidelines from the American Association for the Study of Liver Diseases and European Association for the Study of the Liver recommend HCC screening using ultrasonography with or without α-fetoprotein (AFP) in high-risk patients, including those with cirrhosis.^4,5,6^

The efficacy of HCC screening in patients with cirrhosis is controversial because of the lack of randomized data and the inherent biases of cohort studies, including lead-time and length-time biases.^7^ This controversy was fueled by a case-control study^8^ from the US Veterans Health Administration that failed to find an association between screening and HCC-related mortality. Lead-time bias occurs when screening leads to earlier cancer detection, so the time from diagnosis to death appears longer without an actual difference in mortality. Length-time bias results from screening being more likely to detect slow-growing indolent tumors that are less likely to be fatal, which confers an artificial perception of improved survival.^9^ Although HCC is considered an aggressive cancer with 5-year survival rates less than 20%,^10^ tumor biology is variable. A systematic review^11^ reported an average HCC doubling time of 4 to 5 months, although indolent growth patterns are more commonly observed with nonviral liver disease.

Understanding the true benefit of HCC screening is important for determining its overall value, considering potential physical, financial, and psychological harms.^12,13^ Delayed evaluation of the risk-to-benefit ratio has led to controversies in other cancer screening programs, including prostate cancer, colorectal cancer in older individuals, and breast cancer in younger women. Here, we aimed to characterize the benefits of HCC screening after considering lead-time and length-time biases in a contemporary cohort of patients.

## Methods

### Patient Selection

We performed a retrospective cohort study including patients with cirrhosis from any cause or noncirrhotic chronic hepatitis B virus (HBV) infection with a new diagnosis of HCC between January 2008 and December 2022. Patient recruitment was done at 2 large health systems from the North American Liver Cancer Consortium: UT Southwestern (UTSW) Medical Center (Dallas, Texas) and Parkland Health (Dallas, Texas). UTSW is an academic tertiary-care referral center, and Parkland Health is an integrated safety-net health system.^14,15^ This study was approved by the institutional review board of UTSW Medical Center, with a waiver of informed consent because the study was retrospective and the research involved minimal risk to participants, in accordance with 45 CFR §46. The study follows Strengthening the Reporting of Observational Studies in Epidemiology (STROBE) reporting guidelines for cohort studies.

Patients with HCC were identified using databases of patients seen in multidisciplinary liver tumor clinics at each health system. HCC diagnoses were confirmed to meet the diagnostic criteria per American Association for the Study of Liver Diseases guidelines,^4^ including imaging with Liver Imaging Reporting and Data System category 5 lesions or histological confirmation. We excluded patients with Child Pugh class C cirrhosis or Eastern Cooperative Oncology Group (ECOG) performance status of 2 or higher because screening is not indicated for those populations.

### Data Collection

We collected patient demographics, insurance status, and clinical characteristics such as body mass index (BMI; calculated as weight in kilograms divided by height in meters squared), liver disease cause, ECOG performance status, and Child-Pugh class from the electronic health record (EHR). Race and ethnicity in the EHR were self-reported by patients and were classified for this study as Black, Hispanic, non-Hispanic White, and other (ie, Asian, American Indian or Alaska Native, or unknown).^16^ Data on race and ethnicity are included in this study because of their potential impact on screening outcomes. BMI was classified according to World Health Organization categories.^17^ We classified liver disease cause as hepatitis C virus (HCV), HBV, alcohol-related liver disease (ALD), metabolic dysfunction–associated steatotic liver disease (MASLD), and other, with the following hierarchical algorithm used for patients with multiple causes: (1) HCV, (2) HBV, (3) ALD, (4) other, and (5) MASLD (eg, patients with HCV and ALD were classified as HCV).^18^ Tumor-related characteristics included nodule count, maximum diameter, vascular invasion, and presence of metastases. We captured dates of HCC treatments, including liver transplantation, surgical resection, local ablation, transarterial chemoembolization or radioembolization, stereotactic body radiation therapy, or systemic therapy.

Using manual abstraction from the EHR, including scanned external records, we recorded receipt of AFP measurement and liver imaging (ultrasonography, multiphase computed tomography, or contrast-enhanced magnetic resonance imaging) in the year before HCC diagnosis. We excluded the last imaging study leading to HCC diagnosis, which was performed with diagnostic intent. We classified imaging study indication as screening (eg, to rule out HCC or for cirrhosis), diagnostic (eg, abdominal pain, jaundice, or elevated liver enzymes), other, or unknown. Examinations performed to monitor an indeterminate liver lesion were captured under other within a monitoring of liver mass subcategory. Imaging studies were classified as positive when there was a suspicious liver lesion 1 cm or larger, and AFP was positive when it was greater than or equal to 20 ng/mL (to convert to micrograms per liter, multiply by 1.0).^19^

Screen-detected HCC was defined as (1) prior imaging with screening intent and a positive result, (2) imaging performed for monitoring of a liver lesion (eg, Liver Imaging Reporting and Data System category 3 or 4 lesions)^20^, or (3) positive AFP within the prior 3 months. We further classified screen-detected HCC as that detected by imaging alone, AFP alone, or imaging plus AFP. Patients with false-negative screening results were classified as having screen failure. Non–screen-detected HCCs included those with incidental or symptomatic detection according to the presence of potential HCC-related symptoms.

### Associations With Early Tumor Detection and Treatment

We examined associations of screen detection with clinical outcomes, including tumor stage, curative treatment, and overall survival. Early-stage HCC was assessed using 2 definitions: Barcelona Clinic Liver Cancer (BCLC) stage 0/A and the Milan criteria.^21,22^ Curative treatments were defined as local ablation, resection, or transplantation. Treatment response was assessed using measurements of the enhancing portion of the tumor^23^ and was classified as complete response, partial response, stable disease, progressive disease, or unknown. Objective response rate (ORR) was defined as complete or partial response, whereas the disease-control rate was defined as complete response, partial response, or stable disease as the best response.

### Associations With Overall Survival

Overall survival was captured from HCC diagnosis to death, and patients who were lost to follow-up were censored at their last clinical encounter. Patients were also censored at time of liver transplantation.

Given that the mediating pathway for screening to improve survival would be early tumor detection and curative treatment receipt, we performed stratified analyses by tumor burden and receipt of curative therapy. In the absence of lead-time and length-time biases and residual confounding, differences in mortality between screen-detected and non–screen-detected tumors should be mitigated in these analyses.

### Differential Tumor Growth Patterns

To inform the potential for lead-time and length-time biases, we estimated tumor doubling times (TDTs) among the subset of patients with 2 imaging studies after HCC diagnosis more than 30 days apart without intervening HCC-directed treatment. TDT was calculated using the Schwartz equation, as described elsewhere^25,26^ (eAppendix 1 in Supplement 1). Tumors were categorized as having indolent (TDT ≥365 days), intermediate (TDT, 90-365 days), and rapid (TDT, ≤90 days) growth.

### Statistical Analysis

Data analysis was performed from September to November 2023. We used multivariable Cox proportional hazard analyses to characterize differences in overall survival between screen-detected and non–screen-detected HCC, adjusting for a priori important factors (ie, age, sex, race, BMI, insurance status, liver disease cause, Child Pugh class, and ECOG performance status). Crude and adjusted hazard ratios (HRs) with 95% CIs and 1-year, 3-year, and 5-year survival rates (with 95% CIs) were estimated. Lead-time and length-time bias adjustments were conducted using the parametric method proposed by Duffy and colleagues^24^ (eAppendix 1 in Supplement 1). For all analyses, statistical significance was defined as 2-sided *P* < .05. Analyses were performed using R statistical software version 4.2.1 (R Project for Statistical Computing).

## Results

### Patient Characteristics

A total of 1313 patients (mean [SD] age, 61.7 [9.6] years; 993 male [75.6%]; 739 [56.3%] with BCLC stage 0/A disease) were analyzed. Their characteristics are described in Table 1. The most common causes of HCC were HCV (786 patients [59.9%]), ALD (184 patients [14.0%]), and MASLD (163 patients [12.4%]). The cohort was diverse regarding race and ethnicity (390 Black patients [29.7%], 354 Hispanic patients [27.0%], 477 White patients [36.3%], and 92 patients of other races [7.0%]). Nearly two-thirds (820 patients [62.5%]) had Child-Pugh class A cirrhosis, and 492 patients [37.5%] had Child Pugh class B cirrhosis.

**Table 1.** Characteristics of Patient Population

Characteristic	Patients, No. (%)		
	Total (N = 1313)	With screen-detected HCC (n = 556)	With non–screen-detected HCC (n = 757)
Age at diagnosis, mean (SD), y	61.7 (9.6)	61.9 (9.5)	61.5 (9.6)
Sex			
Male	993 (75.6)	406 (73.0)	587 (77.5)
Female	320 (24.4)	150 (27.0)	170 (22.5)
Race and ethnicity			
Black	390 (29.7)	166 (29.9)	224 (29.5)
Hispanic	354 (27.0)	161 (29.0)	193 (25.5)
White	477 (36.3)	202 (36.3)	275 (36.3)
Other^a^	92 (7.0)	27 (4.9)	65 (8.6)
Cause			
Hepatitis C virus	786 (59.9)	339 (61.0)	447 (59.0)
Hepatitis B virus	89 (6.8)	34 (6.1)	55 (7.3)
Alcohol	184 (14.0)	82 (14.7)	102 (13.5)
Metabolic dysfunction–associated steatotic liver disease	163 (12.4)	73 (13.1)	90 (11.9)
Other	91 (6.9)	28 (2.1)	63 (8.3)
Child Pugh cirrhosis class			
A	820 (62.5)	358 (64.4)	462 (61.0)
B	493 (37.5)	198 (35.6)	295 (39.0)
C	NA	NA	NA
Barcelona Clinic Liver Cancer stage			
0/A	739 (56.3)	393 (70.7)	346 (45.7)
B	261 (19.9)	87 (15.6)	174 (23.0)
C	313 (23.8)	76 (13.7)	237 (31.3)
Insurance status (n = 1305)			
Uninsured	108 (8.3)	32 (5.8)	76 (10.1)
Medicaid	204 (15.6)	87 (15.8)	117 (15.5)
Medicare	439 (33.6)	209 (37.9)	230 (30.5)
Private	248 (19.0)	109 (19.7)	139 (18.5)
Other	306 (23.4)	115 (20.8)	191 (25.4)
Eastern Cooperative Oncology Group performance status			
0	960 (73.1)	439 (79.0)	521 (68.8)
1	353 (26.9)	117 (21.0)	236 (31.2)
Body mass index (n = 1299)^b^			
Underweight (<18.5)	33 (2.5)	10 (1.8)	23 (3.1)
Normal weight (18.5-24.9)	405 (31.2)	156 (28.4)	249 (33.2)
Preobesity (25.0-29.9)	456 (35.1)	192 (34.9)	264 (35.2)
Obesity class I (30.0-34.9)	261 (20.1)	128 (23.3)	133 (17.8)
Obesity class II (35.0-39.9)	79 (6.1)	39 (7.1)	56 (7.5)
Obesity class III (≥40.0)	49 (3.8)	25 (4.5)	24 (3.2)

Abbreviations: HCC, hepatocellular carcinoma; NA, not applicable.

^a^ Other race included Asian, American Indian or Alaska Native, or unknown race and ethnicity.

^b^ Body mass index is calculated as weight in kilograms divided by height in meters squared.

### Patterns of HCC Detection

HCC was screen detected in 556 patients (42.3%) and non–screen detected in 757 patients (57.7%). Characteristics of patients with non–screen-detected HCC are shown in eTable 1 in Supplement 1. Among patients with screen-detected HCC, 248 (44.6%) had detection by imaging alone, 59 (10.6%) had detection by AFP alone, and 249 (44.7%) had detection by both imaging and AFP. The time between a positive screening test and HCC diagnosis was within 6 months for most patients (imaging-detected, 464 patients [93.4%]; AFP-detected, 46 patients [77.9%]). Within the non–screen-detected group, 187 (24.7%) were symptomatic and 570 (75.3%) were incidental. Within the incidental group, most patients (530 patients [78.9%]) had no prior imaging. Only 26 (4.6%) were categorized as experiencing screen failure.

### Early-Stage Detection and Curative Treatment Receipt

Overall, the distribution of HCC was 8.5% (111 patients) BCLC stage 0, 47.8% (628 patients) BCLC stage A, 19.9% (261 patients) BCLC stage B, and 23.8% (313 patients) BCLC stage C; approximately one-half (669 patients [51.1%]) were detected within the Milan criteria. The proportions of early-stage HCC were significantly higher in the screen-detected than non–screen-detected group (BCLC stage 0/A, 393 patients [70.7%] vs 346 patients [45.7%]; risk ratio [RR], 1.54; 95% CI, 1.41-1.70; Milan criteria, 365 patients [66.0%] vs 304 patients [40.2%]; RR, 1.64; 95% CI, 1.48-1.82). The proportions of early-stage HCC across subgroups are reported in eAppendix 2 in Supplement 1.

Overall, 535 patients (40.9%) underwent curative treatment, including liver transplant (123 patients [9.4%]), resection (234 patients [17.9%]), and local ablation (178 patients [13.6%]). Curative treatment was higher in those with screen-detected vs non–screen-detected tumors (283 patients [51.1%] vs 252 patients [33.5%]; RR, 1.52; 95% CI, 1.34-1.74). Among non–screen-detected tumors, curative treatment receipt was higher among patients with incidental tumors than those with symptomatic tumors (204 patients [36.0%] vs 48 patients [25.7%]; RR, 1.40; 95% CI, 1.07-1.83). As anticipated, this association appeared to be mediated by early detection, with similar proportions of patients undergoing curative treatment among those with BCLC stage 0/A vs symptomatic disease (37 patients [50.0%]) and screen-detected HCC (257 patients [65.7%]; RR, 1.31; 95% CI, 1.03-1.67) vs incidental HCC (165 patients [61.1%]; RR, 1.22; 95% CI, 0.95-1.56).

In exploratory analyses, response to first treatment appeared similar between screen-detected, incidental, and symptomatic tumors, with all exhibiting ORR exceeding 80% with curative-intent treatments; ORRs after embolic treatments were similar at 54.8% (RR, 1.22; 95% CI, 0.95-1.56) among screen-detected HCCs and 59.9% (RR, 1.54; 95% 1.08-2.20) among incidental HCC, but lower at 38.9% among HCCs with symptomatic presentations.

### Tumor Growth Patterns and Response to Treatment

**Figure 1.**
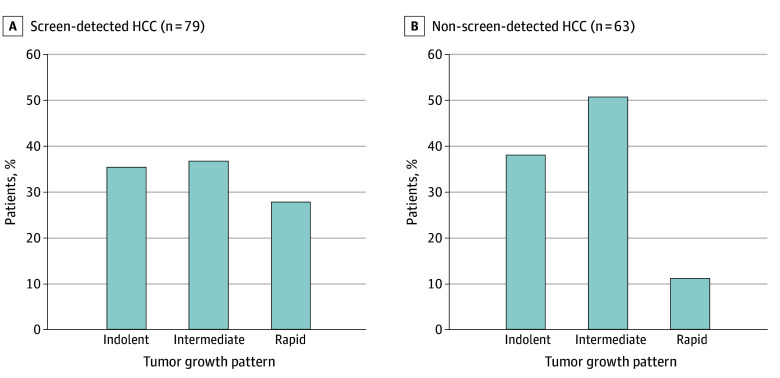
Tumor Growth Patterns Among Early-Stage Group Screen-detected hepatocellular carcinoma (HCC) had a median (IQR) tumor doubling time of 3.8 (2.2-10.7) months vs 5.6 (1.7-11.4) months for non–screen-detected HCC (*P* = .40).

The distribution of TDT for patients with available data (79 screen-detected and 63 non–screen-detected HCC) is demonstrated in Figure 1. Screen-detected HCCs had a median (IQR) TDT of 3.8 (2.2-10.7) months compared with 5.6 (1.7-11.4) months for non–screen-detected HCCs. The proportions of indolent tumors did not significantly differ between the groups (28 patients [35.4%] vs 24 patients [38.1%]; RR, 0.93; 95% CI, 0.60-1.43).

### Overall Survival

**Figure 2.**
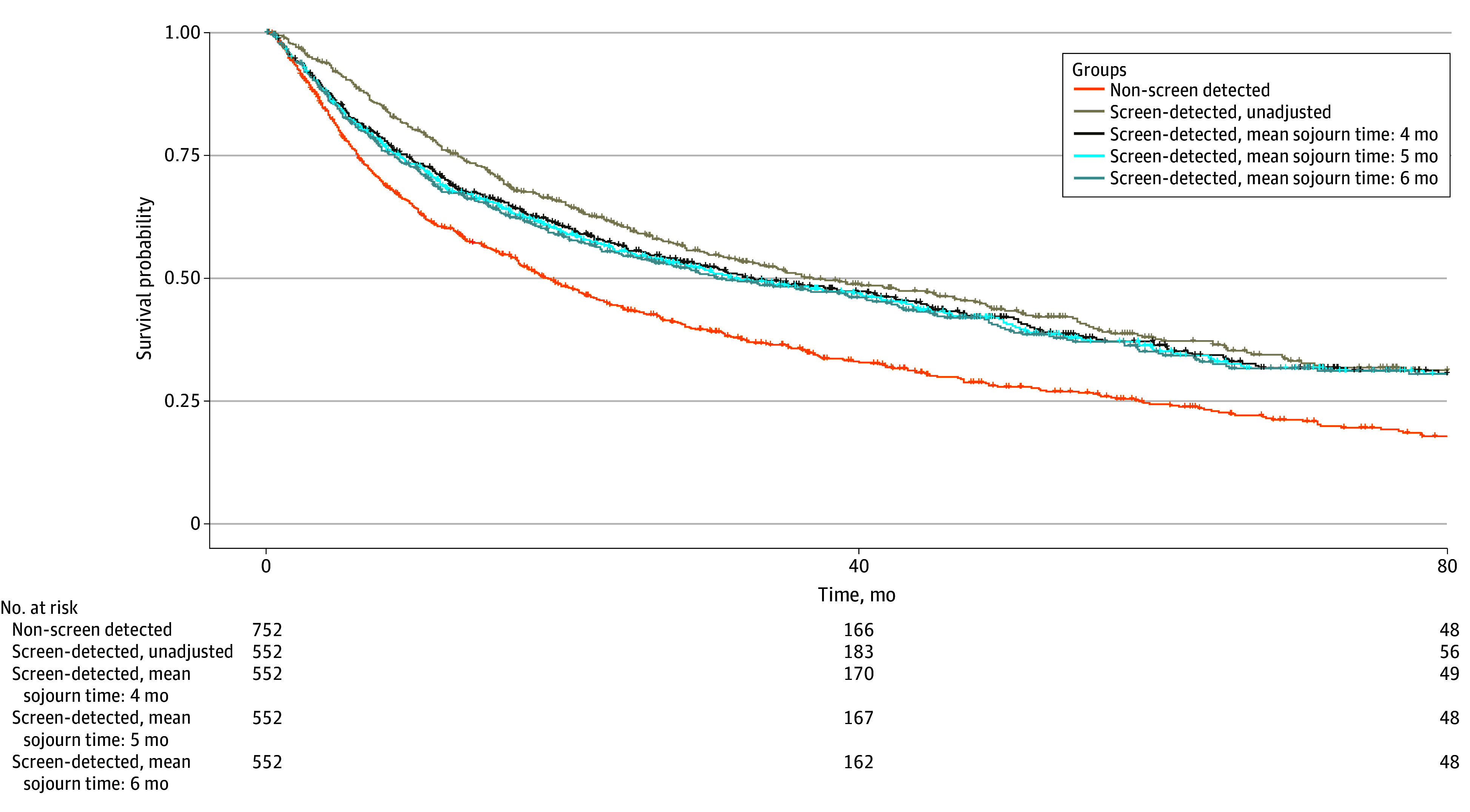
Kaplan-Meier Curves Comparing Patients With Non–Screen-Detected vs Screen-Detected Hepatocellular Carcinoma, With Lead-Time Bias Adjustments for Various Sojourn Times

Median survival was higher in the screen-detected group than in the non–screen-detected group (37.0 months [95% CI, 30.9-47.9 months] vs 19.0 months [95% CI, 16.9-21.9 months]) (Figure 2). Similarly, restricted mean survival time was higher in the screen-detected group (eTable 2 and eFigure in Supplement 1). Among patients with non–screen-detected tumors, survival was higher for those with incidental detection than those with symptomatic presentation (19.9 vs 16.8 months). In multivariable analysis, screen-detected HCC was significantly associated with reduced mortality (HR, 0.65; 95% CI, 0.56-0.75), including after adjustment for curative treatment receipt (HR, 0.75; 95% CI, 0.65-0.87). In exploratory subgroup analyses, imaging detection (HR, 0.67; 95% CI, 0.55-0.80) and AFP detection (HR, 0.69; 95% CI, 0.48-0.98) were both found to be associated with reduced mortality.

After adjusting for lead-time bias (sojourn time 5 months), the median survival of patients with screen-detected HCC decreased to 31.4 months (95% CI, 26.0-42.9 months) (Figure 2). In sensitivity analyses using sojourn times of 4 to 6 months, screen detection remained associated with reduced mortality (Table 2). With a 6-month sojourn time, screen detection was associated with 33% decreased risk of mortality (HR, 0.77; 95% CI, 0.67-0.89). Further adjustment for length-time bias decreased survival estimates, although 3-year and 5-year survival remained longer than for patients with non–screen-detected HCC (Table 3). Patients with screen-detected tumors had 3-year and 5-year survival estimates of 37% and 26%, respectively, compared with 25% and 8%, respectively, for those with non–screen-detected tumors. The association with improved survival remained significant after lead-time bias adjustment among those with HCC detected by imaging, across sojourn times of 4 to 6 months (4 months, HR, 0.75; 95% CI, 0.62-0.90; 5 months, HR, 0.76; 95% CI, 0.63-0.92; 6 months, HR, 0.78; 95% CI, 0.64-0.94); however, there was no association among those with AFP-detected HCC.

**Table 2.** Multivariable Cox Proportional Hazards Model for Association of Screen Detection With Overall Survival, Adjusting for Lead-Time Bias

Variable	HR (95% CI)^a^
Crude (unadjusted)	
Non–screen-detected hepatocellular carcinoma	1 [Reference]
Screen-detected hepatocellular carcinoma	0.65 (0.56-0.75)
Adjusted for lead-time bias	
Mean sojourn time of 4 mo	0.74 (0.64-0.85)
Mean sojourn time of 5 mo	0.75 (0.65-0.87)
Mean sojourn time of 6 mo	0.77 (0.67-0.89)

Abbreviation: HR, hazard ratio.

^a^ Adjusted for age at diagnosis, sex, race, body mass index, insurance status, liver disease etiology, Child Pugh class as a continuous variable, and Eastern Cooperative Oncology Group class.

**Table 3.** Survival Estimates for Patients With HCC, Adjusting for Lead-Time and Length-Time Biases

Variable	Survival rate, % (95% CI)^a^		
	1 y	3 y	5 y
Crude (not adjusted for lead-time or length-time biases)			
Non–screen-detected HCC	63 (51-77)	25 (14-46)	8 (3-26)
Screen-detected HCC	75 (62-90)	46 (29-75)	32 (16-65)
Adjusted for lead time			
Mean sojourn time of 4 mo	68 (54-87)	44 (26-73)	31 (15-65)
Mean sojourn time of 5 mo	66 (51-86)	43 (26-73)	30 (14-64)
Mean sojourn time of 6 mo	65 (50-85)	43 (25-72)	29 (14-63)
Adjusted for lead and length time^b^			
Mean sojourn time of 4 mo	59 (47-76)	38 (23-64)	27 (13-57)
Mean sojourn time of 5 mo	57 (44-75)	37 (23-64)	26 (12-56)
Mean sojourn time of 6 mo	57 (44-74)	37 (22-63)	25 (12-55)

Abbreviation: HCC, hepatocellular carcinoma.

^a^ Adjusted for age at diagnosis, sex, race, body mass index, insurance status, liver disease etiology, Child Pugh class as a continuous variable, and Eastern Cooperative Oncology Group class.

^b^ Length-time adjustment is based on calculated Φ = 0.87.

### Overall Survival, Stratified by Tumor Burden

When stratified by BCLC stage, screen detection was associated with a survival benefit among those with early-stage HCC (BCLC 0/A, HR, 0.79; 95% CI, 0.64-0.97) but not those with larger tumor burden (BCLC B, HR, 1.04; 95% CI, 0.75-1.44; BCLC C, HR, 0.81; 95% CI, 0.59-1.11). Among patients with early-stage HCC, median survival was 56.6 months (95% CI, 51.1-68.9 months) for those with screen-detected HCC vs 48.7 months (95% CI, 40.1-59.4 months) for those with non–screen-detected HCC. Curative treatments appeared to be associated with survival differences among patients with early-stage HCC, because survival was similar between those with screen-detected and non–screen-detected early-stage HCC who received curative treatment (HR, 0.91; 95% CI 0.68-1.23). When stratified by Milan criteria in adjusted analyses, there was no association of HCC screening with survival for either those whose disease fell within the Milan criteria (HR, 0.85; 95% CI, 0.68-1.07) and those whose disease did not (HR, 0.88; 95% CI, 0.72-1.08).

Among those with BCLC stage A HCC, 389 patients (67.0%) had a unifocal HCC between 2 and 5 cm, 51 (8.8%) had a unifocal lesion larger than 5 cm, and 141 (24.3%) had multifocal HCC with each 3 cm or smaller in maximum diameter. Survival did not significantly differ between those with screen-detected and non–screen-detected tumors in all subgroups (unifocal 2-5 cm HCC, HR, 0.97; 95% CI, 0.73-1.29, unifocal >5 cm HCC, HR, 0.70; 95% CI, 0.18-2.69; multifocal ≤3 cm HCC, HR, 0.75; 95% CI, 0.41-1.35) in adjusted models. Among patients with early-stage, non–screen-detected HCC, those with incidental detection had a reduced HR for mortality compared with those with symptomatic presentation, but this difference did not reach statistical significance (HR, 0.84; 95% CI, 0.57-1.22).

## Discussion

In this cohort study, we found that fewer than one-half of at-risk patients with HCC at 2 large US health centers had screen-detected disease. Screen detection was associated with improved early tumor detection, curative treatment receipt, and reduced mortality. The association of screen detection with reduced mortality persisted after adjusting for lead-time and length-time biases across a range of sojourn times.

Prior studies^27,28^ reported a variable association of HCC screening with improved survival after adjustment for lead-time bias. Notably, a case-control study^27^ from the US Veterans Affairs health system failed to find an association of screening with HCC-related mortality. The lack of an association may be related to downstream failures in the cancer care continuum, including underuse of curative treatments in patients with early-stage HCC. Our data reinforce a significant association between screening and improved outcomes, including HCC-related mortality, even after adjusting for lead-time bias across several sojourn times.

Few studies have evaluated the impact of length-time bias, although most suggested that impact was minimal.^29^ We found lower survival estimates for patients with screen-detected HCC after adjusting for length-time bias, although screening continued to have an association with improved survival. We also examined biological differences in tumor growth patterns to inform discussions of length-time bias and overdiagnosis. We noted variation in TDTs and growth patterns, consistent with a prior meta-analysis^11^ that reported an average TDT of 4 to 5 months and approximately one-third of HCCs having indolent growth patterns. Our study adds to this literature by demonstrating similar proportions of indolent tumors between screen-detected and non–screen-detected groups. The similar proportions may relate to the high proportion of incidental detection in the non–screen-detected group, likely because of frequent use of nonscreening imaging among patients with cirrhosis. These data reinforce the impact of length-time bias on screening benefits, and the risk of HCC overdiagnosis is likely small. Overdiagnosis in HCC is further mitigated through guideline recommendations that promote screening in at-risk populations, avoidance of screening in those with high competing risks of mortality (eg, Child-Pugh class C cirrhosis), and strict diagnostic criteria for HCC cases.^30^

Overall, these data highlight the importance of promoting HCC screening implementation in practice. Several studies^31,32^ have demonstrated persistent underuse of screening, related to a combination of patient and practitioner barriers. Although most practitioners believe screening improves early detection and survival, many report a continued need for data evaluating the benefits and harms of HCC screening.

Screening improves overall survival by facilitating early tumor detection. However, the association between screening and survival in our study was only partially mitigated after adjusting for tumor stage. After adjusting for curative treatment, survival differences between patients with screen-detected and non–screen-detected early-stage HCC disappeared. These data reinforce that screening is only the first step in the cancer care continuum,^33^ and patients with early-stage detection must undergo timely curative treatment to have improved survival.^34,35,36^ Efforts are needed to promote patients receiving high-quality care at multidisciplinary, high-volume settings to reduce issues regarding underuse of curative treatments.^34,37,38,39^

### Limitations

Our study has a few limitations. First, it is prone to ascertainment bias for imaging studies not performed in our health systems. This risk was mitigated by a review of outside clinical records. Second, our study is subject to misclassification bias for screen-detected vs non–screen-detected HCC classification, as well as symptomatic vs incidental presentation, although this was minimized by using detailed study indications. Third, our study includes patients from 2 large diverse health systems, but our results may not generalize to broader populations, especially those outside the US. Fourth, there is a risk of residual confounding because other factors, such as recognition of underlying cirrhosis, are needed for HCC screening receipt. Fifth, tumor growth patterns were derived from patients without interval treatment, which may have a higher proportion of indolent tumors, and tumor growth patterns within each patient may vary over time.^40^ We feel these limitations are outweighed by the strengths of the study including its large sample size, diversity regarding race and liver disease cause, and rigorous statistical and biological consideration of lead-time and length-time biases.

## Conclusions

In this cohort study of at-risk patients, lead-time and length-time biases had an impact on survival estimates and should be considered in future studies. However, HCC screening was associated with improved survival after accounting for these biases and remains an important target for multilevel interventions.

## Notes

Supplement 1. **eAppendix 1.** Supplemental Methods **eAppendix 2.** Supplemental Results **eTable 1.** Characteristics of Non–Screen-Detected HCC Patients **eTable 2.** Restricted Mean Survival Times for HCC, Adjusting for Lead Time Bias **eFigure.** Kaplan Meier Curves Comparing Patients With Incidental Versus Symptomatic Non–Screen-Detected HCC
eReferences

Supplement 2.
Data Sharing Statement
