# Supplementary material for: Hepatocellular Carcinoma Screening in a Contemporary Cohort of At-Risk Patients
Source: JAMA Netw Open. 2024 Apr 29;7(4):e248755. doi: 10.1001/jamanetworkopen.2024.8755 (PMC11059036; doi:10.1001/jamanetworkopen.2024.8755)
Supplement: Supplement 1. — eAppendix 1. Supplemental Methods eAppendix 2. Supplemental Results eTable 1. Characteristics of Non–Screen-Detected HCC Patients eTable 2. Restricted Mean Survival Times for HCC, Adjusting for Lead Time Bias eFigure. Kaplan Meier Curves Comparing Patients With Incidental Versus Symptomatic Non–Screen-Detected HCC eReferences [file jamanetwopen-e248755-s001.pdf]

Supplemental Online Content

Daher D, Seif El Dahan K, Rich NE, et al. Hepatocellular carcinoma screening in a contemporary cohort of at-risk patients. *JAMA Netw Open*. 2024;7(4):e248755. doi:10.1001/jamanetworkopen.2024.8755

eAppendix 1. Supplemental Methods
eAppendix 2. Supplemental Results
eTable 1. Characteristics of Non–Screen-Detected HCC Patients
eTable 2. Restricted Mean Survival Times for HCC, Adjusting for Lead Time Bias
eFigure. Kaplan Meier Curves Comparing Patients With Incidental Versus Symptomatic Non–Screen-Detected HCC
eReferences

This supplemental material has been provided by the authors to give readers additional information about their work.

© 2024 Daher D et al. *JAMA Network Open.*

eAppendix 1. Supplemental Methods

Statistical correction of lead-time bias is based on sojourn time which is the period during which the tumor is asymptomatic but screen detectable. The expected additional follow-up time, *s*, due to lead time, is E(s)= $\frac{1-e^{\lambda t}-\lambda t e^{-\lambda t}}{\lambda(1-e^{-\lambda t})}$ for patients with screening known to be dead at time t, and E(s)= $\frac{1-e^{\lambda t}}{\lambda}$ for patients with screening known to be alive at time t. Lead time is corrected by subtracting E(s) from t, the observed survival time. We assumed an HCC sojourn time (1/λ) of 5 months; however, we also performed sensitivity analyses with the mean sojourn times of 4 and 6 months.[11,40] Length-time bias was adjusted based on the population of patients with slow-growing tumors versus aggressive tumors. φ was calculated based on the following formula $\varphi = \frac{p_2\{(\theta q+1-q)(\theta+q(1-\theta))-p_3\theta\}}{p_1\theta(1-p_3)}$, where p_1 is the observed probability of death from symptomatic tumors, p_2 observed probability of death from screen-detected tumors, and p_3 is the observed probability of screen-detected tumors. We considered that 20% of tumors are slow-growing in the length-bias group (1 – *q*) and 0.9 is the relative risk of death from slow-growing tumors, denoted by θ. Multiplying Φ by the survival rate of patients with screen-detected patients, we estimated the length time bias-corrected survival for screen-detected tumors.

Tumor doubling time (TDT) was calculated among HCC cases that were BCLC stages 0 or A. We used spherical measurements for volume $(\frac{4}{3})\pi(\frac{a}{2})^3$ where a is the largest diameter[26]. TDT was calculated using Schwartz' equation[25]. TDT= $\frac{(T-T_0)ln2}{\ln(\frac{V}{V_0})}$, with T = date of last imaging study before treatment, T_0 = date of first imaging study, V = volume of largest tumor in last imaging study before treatment, and V_0 = volume of largest tumor in first imaging study. We included cases with $T\text{-}T_0$ >30 days to ensure sufficient time to observe tumor doubling. For patients with multiple imaging studies, we used the two imaging studies furthest apart in time. We only included measurements from multi-phasic CT or contrast-MRI. For patients with histological diagnosis, we included the imaging study closest to diagnosis for measurements.

eAppendix 2. Supplemental Results

Within the screen-detected group, the proportions of early-stage HCC were similar across subgroups detected by both imaging and AFP (BCLC stage 0/A: 71.9%; Within Milan: 68.4%), by imaging alone (BCLC stage 0/A: 69.8%; Within Milan: 63.3%) and by AFP alone (BCLC stage 0/A: 69.5%; Within Milan: 67.2%). Among non-screen detected patients, the proportions of early-stage HCC appeared higher among those with incidental compared to symptomatic presentation (BCLC stage 0/A: 47.7% vs. 39.6%, RR 1.21, 95% 0.99 – 1.47; Milan Criteria: 42.4% vs. 33.7%, RR, 1.26, 95%CI 1.01 – 1.57)

© 2024 Daher D et al. *JAMA Network Open.*

eTable 1. Characteristics of Non–Screen-Detected HCC Patients

	**Non-screen detected** n=757	**Incidental detection** n= 570	**Symptomatic detection** n=187
Age at diagnosis, mean (SD), y	61.5 (9.6)	62.1 (9.4)	59.8 (10.2)
Sex (% Male)	587 (77.5)	445 (78.1)	142 (75.9)
**Race/Ethnicity, %** (n=757)			
Black	224 (29.5)	153 (26.8)	71 (38.0)
Hispanic	193 (25.5)	146 (25.6)	47 (25.1)
White	275 (36.3)	222 (38.9)	53 (28.3)
Other[a]	65 (8.6)	49 (8.6)	16 (8.6)
**Etiology, %** (n=757)			
HCV	447 (59.0)	322 (56.5)	125 (66.8)
HBV	55 (7.3)	42 (7.4)	13 (7.0)
Alcohol	102 (13.5)	85 (14.9)	17 (9.1)
MASLD	90 (11.9)	74 (13.0)	16 (8.6)
Other	63 (8.3)	47 (8.2)	16 (8.6)
**Child Pugh Class, %** (n=757)			
A	462 (61.0)	349 (61.2)	113 (60.4)
B	295 (39.0)	221 (38.8)	74 (39.6)
C	NA	NA	NA
**BCLC stage, %** (n= 757)			
0/A	346 (45.7)	272 (47.7)	74 (39.6)
B	174 (23.0)	133 (23.3)	41 (21.9)
C	237 (31.3)	165 (28.9)	72 (38.5)
**Insurance status, %** (n=753)			
Uninsured	76 (10.1)	51 (9.0)	25 (13.4)
Medicaid	117 (15.5)	74 (13.0)	43 (23.0)
Medicare	230 (30.5)	183 (32.3)	47 (25.1)
Private	139 (18.5)	114 (20)	25 (13.4)
Other	191 (25.4)	144 (25.4)	47 (25.1)
**ECOG performance status**, % (n= 757)			
0	521 (68.8)	388 (68.1)	133 (71.1)
1	236 (31.2)	182 (31.9)	54 (28.9)

© 2024 Daher D et al. *JAMA Network Open.*

**BMI**, % (n=749)			
Underweight	23 (3.1)	18 (3.2)	5 (2.7)
Normal weight	249 (33.2)	176 (31.3)	73 (39.0)
Preobesity	264 (35.2)	203 (36.1)	61 (32.8)
Obesity class I	133 (17.8)	108 (19.2)	25 (13.4)
Obesity class II	56 (7.5)	41 (7.3)	15 (8.1)
Obesity class III	24 (3.2)	17 (3.1)	7 (3.8)

[a]Other race included Asian, American Indian or Alaska Native, or unknown race/ethnicity.

© 2024 Daher D et al. *JAMA Network Open.*

eTable 2. Restricted Mean Survival Times for HCC, Adjusting for Lead Time Bias

	Restricted mean survival times (SE)[a]
**Crude (unadjusted for lead time bias)**	
Non-screen detected HCC	31.5 (1.15)
Screen-detected HCC	42.8 (1.39)
**Screen-detected HCC adjusted for lead time bias**	
Mean sojourn time of 4 months	40.5 (1.45)
Mean sojourn time of 5 months	40.0 (1.46)
Mean sojourn time of 6 months	39.6 (1.47)

[a]Restricted mean calculated with upper limit of 80 months.

© 2024 Daher D et al. *JAMA Network Open.*

eFigure. Kaplan Meier Curves Comparing Patients With Incidental Versus Symptomatic Non–Screen-Detected HCC

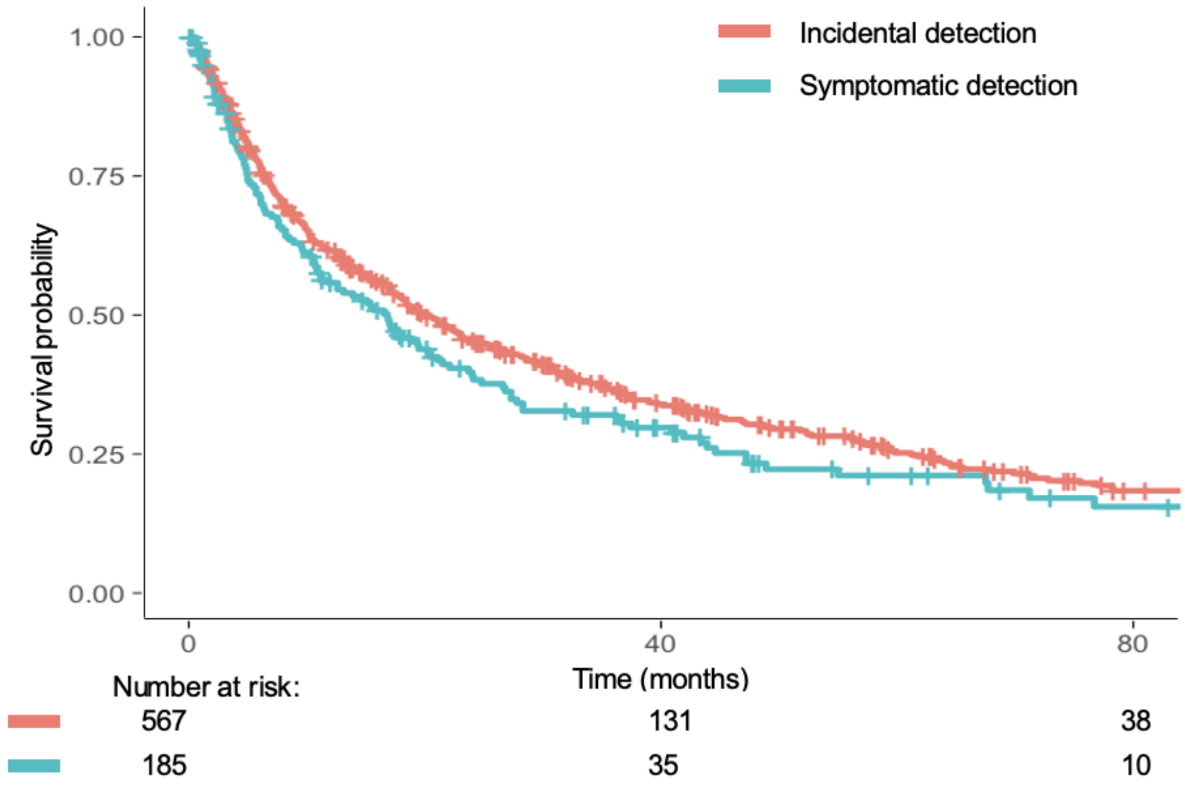

eReferences

1. Nathani P, Gopal P, Rich N, Yopp A, Yokoo T, John B, et al. Hepatocellular carcinoma tumour volume doubling time: a systematic review and meta-analysis. Gut. 2021;70(2):401-7.

2. Schwartz M. A biomathematical approach to clinical tumor growth. Cancer. 1961;14:1272-94.

3. Rich NE, John BV, Parikh ND, Rowe I, Mehta N, Khatri G, et al. Hepatocellular Carcinoma Demonstrates Heterogeneous Growth Patterns in a Multicenter Cohort of Patients With Cirrhosis. Hepatology. 2020;72(5):1654-65.

© 2024 Daher D et al. *JAMA Network Open.*
