# Supplementary material for: Hepatocellular Carcinoma Screening in a Contemporary Cohort of At-Risk Patients
Source: JAMA Netw Open. 2024 Apr 29;7(4):e248755. doi: 10.1001/jamanetworkopen.2024.8755 (PMC11059036; doi:10.1001/jamanetworkopen.2024.8755)
Supplement: Supplement 2. — Data Sharing Statement [file jamanetwopen-e248755-s002.pdf]

Data Sharing Statement

Daher. Hepatocellular Carcinoma Screening in a Contemporary Cohort of At-Risk Patients. *JAMA Netw Open*. Published April 30, 2024. doi:10.1001/jamanetworkopen.2024.8755

Data
Data available: Yes
Data types: Deidentified participant data, Data dictionary
How to access data: The data supporting the findings of this study are available in the article. Data are also available from the corresponding author [AGS] upon reasonable request.
When available: With publication

Supporting Documents
Document types: None

Additional Information
Who can access the data: Data will be available from the corresponding author [AGS] upon reasonable request.
Types of analyses: For any purpose
Mechanisms of data availability: With investigator support, after approval of a proposal and with a signed data access agreement
Any additional restrictions: Requiring a signed data use agreement
